# Adult Presentation of Joubert Syndrome Presenting With Dysphagia: A Case Report

**DOI:** 10.7759/cureus.24226

**Published:** 2022-04-18

**Authors:** Ali Al-Smair, Sara Younes, Osama Saadeh, Ahmad Saadeh, Ahmad Al-Ali

**Affiliations:** 1 Department of Radiology, Medray International Radiology Center, Amman, JOR; 2 Faculty of Medicine, The University of Jordan, Amman, JOR; 3 Public Health, Northeastern Illinois University, Chicago, USA; 4 Department of Radiology, Jordan Ministry of Health, Amman, JOR

**Keywords:** case report, batwing, ciliopathy, molar tooth sign, joubert syndrome

## Abstract

Joubert syndrome (JS) is a rare autosomal recessive disease affecting the cilium, an intracellular organelle. It has a wide spectrum of presentations with the involvement of multiple genes. JS has multiple subtypes that are either pure JS or JS with other organ involvement such as the kidneys, liver, and others. However, all subtypes share the involvement of the cerebellar peduncles and the brainstem, which presents as “a molar tooth sign” on magnetic resonance imaging, hypotonia, and intellectual disability. It has a higher prevalence among children with few able to survive to adulthood. Unfortunately, survivors live with debilitating comorbidities. Here, we present the case of a 20-year-old patient who presented with a new onset of dysphagia that led to a diagnosis of JS.

## Introduction

Joubert syndrome (JS) is a rare autosomal recessive disease involving multiple genes. The prevalence of this congenital disease is 0.5 per 100,000, which increases to 1.8 per 100,000 in children [1]. It is part of a larger group of disorders called ciliopathies caused by the dysfunction of a subcellular organelle called cilium. It is characterized by cerebellar vermis hypoplasia and a molar tooth sign, a brain stem malformation that can be seen on magnetic resonance imaging (MRI) [2]. Although some patients survive to adulthood with comorbidities including motor and intellectual disability, many others do not survive. In general, it presents in childhood with hypotonia, ataxia, tachypnea, and oculomotor apraxia [3]. However, it has a wide range of presentations depending on the subtype present in the patient [2]. It can present as a pure neurological type or as a more severe type with renal, hepatic, orofacial, ocular, and skeletal involvement [1]. Here, we present the case of a 20-year-old patient who presented with a new onset of dysphagia that led to a diagnosis of JS.

## Case presentation

**Figure 1:**
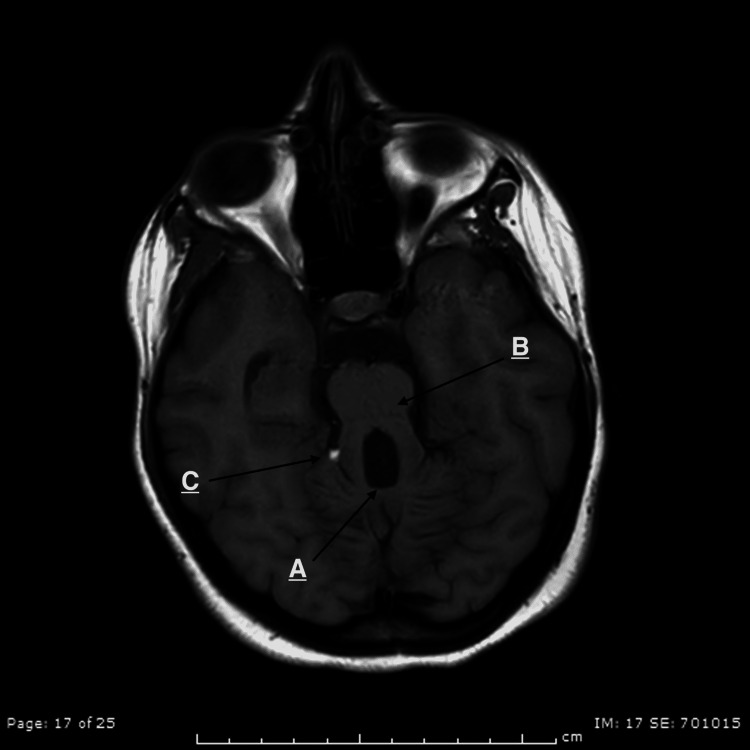
Axial T1 image showing (A) a hypoplastic cerebellar vermis, (B) elongated superior cerebellar peduncles with (A, B) characteristic molar tooth sign. (C) An Incidental right quadrigeminal cistern lipoma.

A 20-year-old patient presented with complaints of dysphagia for the last few months. It occurred while swallowing when food tended to get stuck in her throat. It had started five months before the presentation, and it was progressive as it started with solids, followed by both solids and liquids. She started to lose weight during that period due to decreased oral intake. She was diagnosed with hypotonia in her neonatal period. She was also diagnosed with moderate intellectual disability in her childhood, had a deficient vocabulary, and was living with her family. She was fully vaccinated, with no family history of similar complaints, other gastrointestinal tract disorders, neurological diseases, and neuromuscular diseases. On physical examination, she was alert, oriented, conscious, and vitally stable. She had no signs of facial nerve palsy (no face or lid drop, mouth angle drop, and loss of wrinkles). The trigeminal nerve test was inconclusive (intact sensations, jaw movement, and mastication). Oral cavity examination was normal, apart from absent gag reflex. Other cranial nerves were examined and were normal. General neurological examination showed decreased upper limb tone, normal sensation, and decreased proprioception bilaterally. Lower limb examinations showed decreased tone, normal sensation, decreased proprioception, and truncal and gait ataxia. A central cause of dysphagia was suspected due to the clinical picture and previous history, and an MRI was performed. It showed a hypoplastic cerebellar vermis with elongated superior cerebellar peduncles showing the characteristic molar tooth sign and an incidental right quadrigeminal cistern lipoma (Figure 1).

**Figure 2:**
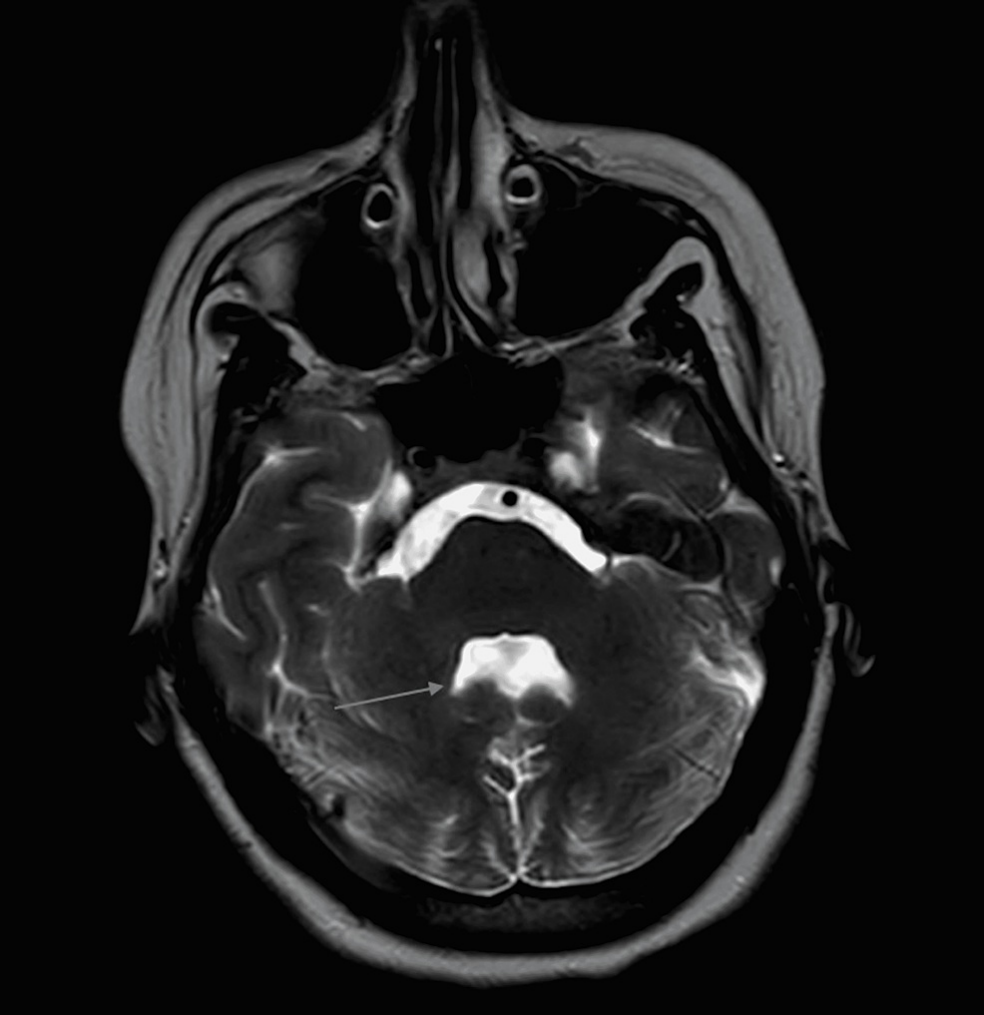
Axial T2 image showing the batwing shape of the fourth ventricle.

Axial T2 image showed the batwing appearance of the fourth ventricle which occurs secondary to vermis hypoplasia (Figure 2).

After considering childhood history, physical examination, and radiological findings, the diagnosis of JS was confirmed.

## Discussion

JS is a rare inherited congenital cerebellar ataxia with an estimated prevalence of 0.5 in 100,000 in the overall population and 1.8 per 100,000 in children [1]. It was first described by Dr. Marie Joubert in 1968 in four siblings with agenesis of cerebellar vermis presenting as episodic hyperpnea, abnormal eye movements, ataxia, and intellectual disability [3].

The congenital brain malformation involves the absence of cerebellar vermis, a structure responsible for posture and locomotion, and abnormal decussation of axons in corticospinal tracts and superior cerebellar peduncles. Thus, JS patients cannot walk because of severe clumsiness, ataxia, and mirror movements [4]. The cerebellum has been shown to be involved in regulating intellect and emotions [5], which can explain the neurocognitive and behavioral disorders in JS patients.

Although the clinical presentation of JS is heterogeneous, the diagnosis of classic or pure JS is based on the presence of the following three: molar tooth sign on MRI, hypotonia in infancy with later development of ataxia, and developmental delay or intellectual disabilities. Our case fulfilled all of the above criteria. An essential aspect of diagnosing JS is its associated radiological findings. The term molar tooth sign is used to describe the findings on MRI resulting from vermis hypoplasia, long thick superior cerebellar peduncles, and a deep interpeduncular fossa [6,7]. In our case, all of the above were found on MRI, and the batwing appearance of the fourth ventricle resulted from severe hypoplasia of the vermis [8].

The mean age of diagnosis in JS is typically 33 months [9]. However, our case was diagnosed at the age of 20 with an initial presentation of dysphagia, and it was only retrospectively found that the patient had hypotonia and intellectual disability in her childhood. To our knowledge, none of the previous reports from Jordan described such a late presentation of JS.

JS is an autosomal recessive disorder with gene heterogenicity. Among 23 genetic mutations associated with JS and related disorders, only 15 have been confirmed in Arab patients. However, the only mutation shown in JS patients among Jordanians was in *transmembrane protein 237* (*TMEM237*), a gene involved in the regulation of ciliogenesis and WNT signaling [10].

## Conclusions

JS is a rare autosomal recessive disease with variable presentation. The mean age at diagnosis is 33 months and a few can survive to adulthood with debilitating comorbidities. It classically presents with hypotonia, developmental delay, and a molar tooth sign on MRI. We have reported the case of an adult with JS in Jordan who presented with dysphagia. This case emphasizes conducting further genetic studies among Jordanian patients with JS and related disorders highlighting the *TMEM237* gene. We have also presented interesting radiological findings seen in such cases.
